# Associations among living alone, social support and social activity in older adults

**DOI:** 10.3934/publichealth.2020042

**Published:** 2020-07-14

**Authors:** Elizabeth Chan, Elizabeth Procter-Gray, Linda Churchill, Jie Cheng, Rachel Siden, Annabella Aguirre, Wenjun Li

**Affiliations:** Department of Medicine, University of Massachusetts Medical School, Worcester, MA, USA

**Keywords:** social support, exercise, social networks, successful aging

## Abstract

**Objectives:**

We examined cross-sectional associations of living alone with social isolation among community-dwelling older adults in Worcester County, Massachusetts, USA.

**Methods:**

Four hundred participants 65 years old and older were recruited in community group settings or by direct mail. Participants were queried for living status, social support, frequency of social activity, sociodemographic and lifestyle factors, and health conditions. Social isolation was assessed by lack of social support and decreased frequency of social activity. Physical activity (PA) was measured via an accelerometer and global positioning system (GPS), which was worn by the participant, for at least 7 consecutive days.

**Results:**

Participants living alone (N = 110) had less social support than those living with others (N = 290) (p < 0.001) but did not differ significantly in the frequency of their social activities. Group-setting recruitment was strongly associated with greater social activity (p < 0.001). Less social support was independently associated with a less-than-high-school education (p = 0.001), higher CES-D depression score (p < 0.001) and lower PA (p = 0.003). Less social activity was independently associated with a less-than-high-school education (p = 0.007) and annual income less than $50,000 (p = 0.01).

**Discussion:**

Older adults who are socioeconomically disadvantaged, have less social support, and who live alone are more likely to be socially isolated and may benefit from continuation of low-cost social activities and increased social support inside the home. Identifying correlates of social isolation may inform future interventions.

## 1. Introduction

Of the 49.2 million adults aged 65 and over in the United States, about 26% live alone [1]. Living alone and social isolation have been shown to be independently associated with a higher risk of mortality [2],[3]. They are important determinants of healthy aging and longevity.

Social isolation refers to a decrease in social relationships and social network size, and lack of social engagement, which includes social activities and social interactions with others [3]–[5]. Social isolation has been shown to be associated with poor cardiovascular disease prognosis [6], cognitive decline [7], decreased immune function [8] and worse physical and mental health [9]. The risk of social isolation has also been associated with older age, lower education, poorer mental health, and difficulties with activities of daily living [10]. Social isolation can further be divided into objective and subjective isolation. Subjective isolation focuses on how one perceives their experience including their sense of loneliness, quality of social networks and social support. Objective isolation is quantifiable focusing on one's type and amount of social support, frequency of participation in social activities, and size of social networks among other factors [11]. Both subjective and objective isolation are important to assess when studying social isolation [11].

Studies have reported conflicting results on living alone as a risk for poorer health. While some studies reported living alone was not a predictor for higher risks for morbidity or hospitalization [12],[13], other studies showed it was [14]. Therefore, continued investigation of living alone in older adults is necessary.

In addition to the well-known health benefits of physical activity (PA) [15], participation in social activities has been shown to increase survival in older adults even among the least physically active [16]. The benefit may be through providing older adults with meaningful social roles [16]. Participating in social activities has also been shown to reduce depression in socially isolated people [17].

Social isolation involves multiple factors including psychological, social and physical factors and has a higher tendency to develop when risk factors overcome protective factors [11]. Risk factors include but are not limited to living alone, low income, inadequate social support, and mental health challenges (e.g. depression and anxiety) while protective factors include strong mental health, absence of physical impairment, and access to social support [11].

The goal of this study was to identify the factors associated with social isolation in older adults, assessed by quantity and quality of social support, engagement in social activities, and living status.

## 2. Methods

### 2.1. Study area and participant recruitment

Community-dwelling adults 65 years old and older were recruited from urban, suburban and rural neighborhoods in Worcester County, Massachusetts, USA between 2012 and 2018. Worcester County is a metropolitan area in Central Massachusetts and has a socioeconomically and geographically diverse older population. The participants were recruited using an area-based sampling strategy to ensure the representativeness of the sample. Our sampling frame was based on the latest town census of residents. US census-based data on minority concentrations at census tract-level was used to over-sample racial/ethnic minorities to increase diversity and representativeness of the cohort. Census tracts were also used to ensure that various neighborhood and community settings were adequately represented. To increase the diversity and representativeness of the cohort, rural residents were also over-sampled. Recruitment took place via group setting and direct mail campaign.

In group settings, participants were recruited to the study via presentations at senior centers, veterans' organizations, and retirement villages. Promotional materials and interest surveys were tailored to each site. Direct mail including letters and interest surveys were sent to randomly selected households with older adults, stratified by urban, suburban, and rural zip codes. Potential participants' names and addresses were obtained through the latest town census.

Once potential participants expressed interest in the study, the staff explained the study, determined eligibility, and mailed a consent form to the potential participant. The study excluded persons who were under age 65, unable to consent, unable to speak English, not living independently such as in assisted living or nursing facilities, unable to walk with or without assistive devices, unable to perform all study-related activities independently or with a designated caregiver, or had 4 or more errors when answering the Short Portable Mental Status Questionnaire (SPMSQ) [18].

If eligible, participants were sent a consent form, and a one-on-one visit with a study team member was scheduled at the participant's home, senior center, or at the University of Massachusetts Medical School. At the visit, the consent form was reviewed, participants were given the opportunity to have their questions answered, and then signed the consent form. Participants completed a set of surveys and physical measurements as detailed below.

The study protocol was approved by the University of Massachusetts Medical School Institutional Review Board (Docket #: H-14793 and H00015047).

### 2.2. Outcomes

Social support was assessed using the Lifestyle Questionnaire (Women's Health Initiative, 2011), which included 9 questions regarding the availability of social support in various circumstances. Each question was answered with response ranging from 0 to 4 points: 0 = None of the time; 1 = A little of the time; 2 = Some of the time; 3 = Most of the time; and 4 = All of the time. A mean social support score, used in the analyses below, was calculated for any participant who answered at least 5 of the 9 questions (N = 400). The 9 items incorporated into the score had a Cronbach's alpha statistic for scale reliability of 0.93. A factor analysis revealed that 65.6% of the variation loaded in the first factor. The first factor was used as a scale for further analysis.

Social activities were measured by asking participants how often they do each of the following activities: eat out of the house; go shopping; go to a cultural event such as a movie, concert, play or lecture; meet with family or friends who do not live with them; communicate with family or friends by phone or email; and go to church or other religious center. Answers were recorded as frequencies per month, and their sum, the monthly frequency of social activities, was used as an outcome for analyses.

### 2.3. Participant characteristics and covariables

Participant characteristics were self-reported using both structured questionnaires that were designed by this study and also standardized instruments mentioned above. Participants who lived alone were identified from a personal information questionnaire, by their response to the question, “Do you currently live with anyone?”

Each participant answered questions about their sociodemographic characteristics, physical and mental health conditions, lower extremity symptoms and problems, history of falls and fall injuries, health care utilization in the past year, and lifestyle behaviors. Physical limitations were further assessed with the Activities of Daily Living survey [19]. Participants' psychosocial factors were self-reported using survey questionnaires. Participants were assessed for risk of clinical depression via the CES-D Depression Scale [20]. Anxiety was measured via the Beck Anxiety Inventory [21].

Body mass index was calculated from weight and height measured by the interviewer and self-reported. PA was measured via accelerometer (ActiGraph GT3X-Plus), which was worn by the participant during waking hours for at least 7 consecutive days. The accelerometer measured the number of steps during each minute of wear-time, which was used to calculate the (12-hour) daily mean number of steps for each participant.

### 2.4. Statistical analysis

Participant characteristics were summarized in total and by their home living situation: living with other(s) vs. living alone. Between-group differences in sociodemographic, physical and mental health, and lifestyle factors were evaluated using Chi-squared tests for percentages or Wilcoxon rank-sum tests for continuous variables. The non-parametric Wilcoxon test was chosen because several of the continuous characteristic variables were not normally distributed.

For each social support item queried, we calculated the percent of participants reporting support “most” or “all” the time and compared these percentages by living situation using Chi-squared tests. Mean social support scores as well as mean monthly frequencies of social activities were also reported by living situation, and the Wilcoxon rank-sum test was used to evaluate the differences.

We estimated the associations of the social support score and monthly frequency of social activities with 26 personal characteristics, first, using unadjusted linear regression models. Assumptions of linearity were investigated and, if violated, the predictor variable was categorized. We then constructed a multivariable linear regression model including all characteristics that retained a robust association with either outcome while adjusting for all other covariables. Although the two outcomes had a strong association with each other, they were omitted as covariates from the multivariable models. Age and sex were included in the models a priori, and the other factors retained were education level, annual household income, living alone, depression score, PA (step count), BMI, and recruitment method.

The statistical package used in all analyses was Stata 14.2 (Stata Corp., College Station, TX, USA). P-values <0.05 were regarded as statistically significant.

## 3. Results

### 3.1. Characteristics of participants by living situation

**Table 1.** Characteristics of participants by living situation (mean ± SD or %).

	All (N = 400)	Not living alone (N = 290)	Living alone (N = 110)	P-value^a^ for diff.
Sociodemographic				
Age (years)	74.1 ± 6.3	73.1 ± 5.9	76.8 ± 6.8	<0.001
Range	65–95	65–92	65–95	
Age ≥80 (%)	19.8	14.8	32.7	<0.001
Sex (% female)	52.3	45.9	69.1	<0.001
Race (% White)	90.2	89.3	92.7	0.30
Years of education	15.3 ± 2.8	15.4 ± 2.8	15.0 ± 2.6	0.14
Annual income <$50,000	36.8	26.0	66.0	<0.001
Married or living with partner	63.7	86.9	1.8	<0.001
Living alone	27.5	-	-	-
Physical health				
Self-rated health fair-to-poor	4.5	2.9	9.1	0.011
Number of comorbid conditions^b^	1.4 ± 1.2	1.3 ± 1.1	1.6 ± 1.2	0.013
Heart or circulatory condition	49.8	48.3	53.6	0.34
Diabetes	11.6	10.4	14.8	0.22
Respiratory disease	13.4	13.7	12.4	0.73
Cancer	18.8	16.6	25.0	0.06
Rheumatoid arthritis	5.7	5.0	7.5	0.34
Osteoarthritis	24.3	21.7	30.9	0.06
Osteoporosis	15.1	13.3	20.2	0.09
Poor vision	1.0	1.4	0.0	0.22
Physical limitations (ADL)	7.5	5.5	12.7	0.015
Mental health				
CESD Depression Scale	5.8 ± 5.8	5.4 ± 5.8	6.9 ± 5.8	0.003
Beck Anxiety Inventory	4.5 ± 5.2	4.3 ± 5.0	5.2 ± 5.9	0.18
Lifestyle				
Mean step count per 12-hour day	4373 ± 2238	4620 ± 2235	3723 ± 2122	<0.001
BMI (kg/m^2^)	27.2 ± 5.0	27.4 ± 4.9	26.8 ± 5.2	0.25
Weekly frequency of alcohol	2.1 ± 2.5	2.3 ± 2.5	1.6 ± 2.3	0.001
Current smoker	4.8	4.9	4.6	0.90
Current motor vehicle operator	99.2	100.0	97.1	0.004
Paid or volunteer employment	22.3	22.8	20.9	0.68

Note: SD = standard deviation; ADL = activities of daily living. The Sociodemographic characteristics are from the Personal Health from Women's Health Initiative (WHI) (2006); Physical health is from the Lifestyle Questionnaire from WHI (2011) and Medical History from WHI (2005); The Beck Anxiety Inventory is from Beck et al. (1988); The CESD Depression Scale is from Radloff (1977); Lifestyle is from the Lifestyle Questionnaire from WHI (2011) and Personal Habits from WHI (2005). ^a^ P-values estimated from chi-square tests for categorical measures and Wilcoxon rank-sum sum tests for continuous measures. ^b^ Number of comorbidities from the following: heart or circulatory conditions (stroke, ischemic attack, high blood pressure); respiratory (asthma, COPD); cancer or malignant tumor; rheumatoid arthritis (not including rheumatism); diabetes; osteoporosis; and osteoarthritis.

Participants included 290 (72.5%) participants who live with others and 110 (27.5%) participants who live alone. Overall, they had a mean (SD) age of 74.1 (6.3) years and 15.3 (2.8) years of education, and 90.2% identified as White (Table 1). Compared to participants not living alone, those living alone were more likely to be older, women, unmarried, and have an annual income <$50,000, lower self-rated health, higher number of comorbid conditions, greater physical limitations, higher score on the depression scale, lower mean step count per 12-hour day, lower mean frequency of alcohol consumption, and lower likelihood of being a current motor vehicle operator (Table 1).

### 3.2. Social support

As shown in Table 2, participants living alone had significantly less social support, overall and in 6 out of 9 specific types of support. Participants living alone had less support with having someone to take them to the doctor when needed, help them understand a problem, help with daily chores if sick, share their private worries and fears, do something fun with, and love them and make them feel wanted.

**Table 2.** Percentage of support available “most” or “all of the time” for 9 items, mean social support score, and frequency of selected social activities, by living situation.

	All (N = 400) %	Not living alone (N = 290) %	Living alone (N = 110) %	Diff.	P^a^ for diff.
Social support item (having someone to… )					
Listen when I need to talk	85.7	87.2	81.8	5.4	0.17
Give me advice about a problem	79.3	80.7	75.5	5.2	0.25
Take me to the doctor when needed	86.4	89.5	78.2	11.3	0.003
Have a good time with	84.5	86.6	79.1	7.5	0.07
Help me understand a problem	82.6	85.0	76.4	8.6	0.043
Help with daily chores if I am sick	76.6	83.0	59.6	23.4	<0.001
Share my private worries and fears	74.4	77.9	65.1	12.8	0.010
Do something fun with	81.7	84.4	74.3	10.1	0.020
Love me and make me feel wanted	84.8	92.6	64.5	28.1	<0.001
Social support score^b^ (mean ± SD)	3.2 ± 0.8	3.3 ± 0.8	3.0 ± 0.9	0.3	<0.001
Monthly frequency of activities					
Eat out	3.6 ± 1.7	3.7 ± 1.6	3.2 ± 1.8	0.5	0.009
Go shopping	4.2 ± 1.3	4.2 ± 1.4	4.4 ± 1.2	0.5	0.22
Go to cultural event (play, concert, etc.)	1.5 ± 1.5	1.5 ± 1.5	1.6 ± 1.7	−0.1	0.82
Visit with friends or family	3.5 ± 1.6	3.6 ± 1.5	3.5 ± 1.6	0.1	0.59
Phone or email family or friends	4.4 ± 1.1	4.4 ± 1.1	4.5 ± 1.1	−0.1	0.53
Go to church or other religious center	2.4 ± 2.3	2.3 ± 2.3	2.5 ± 2.4	−0.2	0.50
Sum of the above activity frequencies	19.5 ± 5.0	19.5 ± 5.0	19.5 ± 5.0	0	0.92

Note: SD = standard deviation. Social support item and monthly frequency of activities from the Lifestyle Questionnaire from WHI (2011). ^a^ P-values estimated from chi-square tests for percentages and Wilcoxon rank-sum sum tests for means. ^b^ Nine social support questions were averaged for this score. Questions were of the form, “How often is each of the following kinds of support available to you when you need it?” Answers: 0 points = “None of the time”; 1 = “A little of the time”; 2 = “Some of the time”; 3 = “Most of the time”; and 4 = “All of the time”.

Overall, social support scores were generally high, with only 10.5% of participants having a mean score of 2 or less, indicating that support was available only “some” of the time, and only 2.3% averaging “little” of the time. Table 3 shows associations of the social support score with a number of personal factors. Without covariate adjustment, lower social support was associated with older age, education less than high school education, being unmarried, living alone, rheumatoid arthritis, physical limitations, higher CES-D depression score, higher anxiety level, low level of PA, current smoking, and less frequent social activities. After multivariable adjustments, less-than-high-school education, living alone, higher CES-D depression score, and lower PA (<2000 steps per 12-hour day) were all independently associated with lower social support score.

### 3.3. Social activities

In contrast with social support, social activity frequency did not differ greatly between participants living alone and living with others, except that participants who lived alone ate out less frequently (Table 2). The mean frequency of all social activities combined was equal for the two groups. Monthly frequency of social activity was negatively associated with less-than-high-school education, higher CES-D depression score, lower BMI, lower social support score, and direct mail recruitment method (Table 3). After adjustment for the other characteristics, less-than-high-school education, lower income, lower BMI, and direct mail recruitment method were independently associated with a lower frequency of attending social activities. Men tended to have less social activity than women (p = 0.05).

**Table 3.** Associations of personal characteristics with mean social support score^a^ and with monthly frequency of selected social activities, unadjusted and adjusted^b^.

	Association with social support score				Association with monthly frequency of social activities			
	Unadjusted		Adjusted^b^		Unadjusted		Adjusted^b^	
	Coef.	P	Coef.	P	Coef.	P	Coef.	P
Sociodemographic								
Age (per year)	−0.02	0.006	0.00	0.96	−0.06	0.11	0.03	0.53
Female	0.04	0.61	0.12	0.18	0.87	0.08	1.09	0.05
White race	−0.12	0.40			0.19	0.82		
Education less than high school graduation	−1.05	<0.001	−0.95	0.001	−5.26	0.003	−5.00	0.007
Annual income <$50,000	−0.07	0.46	0.14	0.15	−0.89	0.10	−1.58	0.01
Married or living with partner	0.35	<0.001			0.33	0.52		
Living alone	−0.39	<0.001	−0.45	<0.001	0.00	0.99	0.70	0.30
Physical health								
Self-rated health fair-to-poor	−0.39	0.06			−2.14	0.09		
Num. of comorbid conditions^c^	−0.06	0.08			0.01	0.95		
Heart or circulatory condition	−0.03	0.70			0.33	0.52		
Diabetes	−0.15	0.24			0.19	0.81		
Respiratory disease	−0.07	0.55			−0.39	0.60		
Cancer	0.04	0.68			−0.08	0.91		
Rheumatoid arthritis	−0.42	0.02			−1.10	0.32		
Osteoarthritis	−0.14	0.16			−0.12	0.84		
Osteoporosis	−0.13	0.25			0.21	0.76		
Poor vision	0.01	0.98			−0.27	0.92		
Physical limitations (ADL)	−0.43	0.006			−0.48	0.61		
Mental health								
CES-D depression score	−0.04	<0.001	−0.03	<0.001	−0.11	0.008	−0.06	0.14
Beck anxiety inventory	−0.03	<0.001			−0.08	0.10		
Lifestyle								
PA: >2000 steps per 12-hr day	0.45	<0.001	0.41	0.003	1.31	0.06	1.61	0.06
BMI (kg/m^2^, per unit)	0.00	0.81	0.00	0.74	0.11	0.04	0.14	0.01
Weekly frequency of alcohol	0.02	0.18			0.02	0.85		
Current smoker	−0.40	0.04			−1.87	0.11		
Current motor vehicle operator	−0.02	0.96			−2.48	0.39		
Paid or volunteer employment	−0.10	0.33			0.03	0.96		
Social Activities								
Monthly freq. of social activities^d^	0.03	<0.001						
Social support score^a^					1.27	<0.001		
Recruitment method								
Group setting (vs. direct mail)	0.12	0.15	0.00	0.99	2.09	<0.001	2.38	<0.001

Note: SD = standard deviation; coef. = linear regression coefficient of the characteristic as predictor of outcome (mean social capital score or monthly activity frequency); freq. = frequency; PA = physical activity; BMI = body mass index. ^a^ Nine social support questions were averaged for this score. Questions were of the form, “How often is each of the following kinds of support available to you when you need it?” Answers ranged from 0 points, “None of the time”, to 4 points, “All of the time”. ^b^ Linear regression model containing the following covariates: age, sex, education, annual household income, living alone, CESD depression score, daily step count, BMI, and recruitment method. ^c^ Number of comorbidities from the following: heart or circulatory conditions (stroke, ischemic attack, high blood pressure); respiratory (asthma, COPD); cancer or malignant tumor; rheumatoid arthritis (not including rheumatism); diabetes; osteoporosis; and osteoarthritis. ^d^ Sum of monthly frequencies of eating out, going shopping, attending a cultural event (play, concert, etc.), meeting with family or friends not of the household, communicating socially by email or phone, and going to church or other religious center.

## 4. Discussion

This cross-sectional study examined the patterns and correlates of social isolation in community-dwelling older adults in the northeastern part of the United States. We identify specific factors associated with social isolation in older adults and emphasize the importance of recruitment method in selecting participants for future interventions.

Living situation may strongly influence social support in older adults. Our analysis found that living alone was associated with lower social support but not with frequency of social activities. Social support can be further divided into instrumental support (e.g. transportation, financial), informational support (e.g. advice), and emotional support (e.g. expressions of caring and empathy) [11]. The participants living alone had, on average, less instrumental, information and emotional support overall in 6 out of 9 specific areas compared to those who live with others. Interestingly, fewer differences in frequency of attending social activities were observed between participants living alone and those not, even after adjusting for personal and lifestyle factors.

This finding suggests that interventions to alleviate social isolation among older adults who live alone should include instrumental, informational and emotional support accessible when they are inside their homes. Those living alone may have circles of friends and family to participate in activities with, however, they may need more support with, for example, household chores, local transportation, or just someone to talk to about their worries and ask for advice. Studies have shown that older adults who live alone benefit from having a reliable person to contact in emergency situations [12]. Therefore, focusing on providing social support with household and emotional needs may be more important than the activities they attend outside the house.

Furthermore, participants living alone reported poorer self-rated health, more comorbid medical conditions, more physical limitations, and a higher level of depression, along with lower social support. There was also a decreased number of participants who were current motor vehicle operators among those living alone. Lack of transportation compounded with physical impairment and chronic illness are risk factors for social isolation and can lead to decreased social contacts [11]. Therefore, healthcare providers may need to be more attentive to the physical and mental health of older adults living alone during office visits [10].

Better understanding the correlates or predictors of social isolation may inform better designed intervention to promote healthy aging. Our study found a number of personal factors independently associated with lower social support, including less than high school education, living alone, higher CES-D depression score, and inadequate PA. Interestingly, when these other variables were taken into account, neither age nor physical limitations were associated with social support. This may indicate that the decline in social support with age and physical limitations that was seen in unadjusted models could be offset by certain modifiable factors, suggesting the opportunities for interventions.

We found that those with less PA had a lower social support score. Those factors combined can put older adults at greater risk of social isolation especially when one lacks physical mobility but also instrumental support in terms of transportation [11]. Additionally, PA has been shown to be associated with a higher number of close friends and higher social support [22],[23]. Older adults with increased social support may have greater support to be physically active. On the other hand, older adults who are physically active, especially in group exercise classes, may develop more social support through increased social contacts. It is hard to determine causation between social support and PA, which calls for more longitudinal studies exploring this area [23].

We found that depression was independently associated with decreased social support, as has been reported in previous research [24]. In addition, subjective social support has been found to be a predictor of depressive symptoms in both men and women, and an important predictor of late-life depression only for men [25],[26]. People who have depression have reported having more negative social interactions as well, which may be why we see less frequency of social support in participants who are depressed [27]. Longitudinal analysis is needed to better understand the dynamic relationships between depression and social isolation.

Education level may also influence the level of social support in older adults. Higher levels of education have been linked to better health outcomes due to the individual's greater sense of capability and self-efficacy, which therefore encourages a healthy lifestyle and avoidance of risky habits [28]. Lower education level has been found to be associated with both social isolation and poorer cognitive function [3],[29]. Education level may be a distinguishing factor that could be used to identify older adults in need of social support.

Education, in addition to its association with social support, was also independently associated with the frequency of social activities, as were income less than <$50,000, lower BMI and direct mail recruitment method. Personal income has been shown to be important in social functioning [30]. Those who are poorer and have less education have been observed to have smaller friendship networks, which may explain why we see less participation in social activities as well [22]. If confirmed, this finding would support the development and continuation of free or low-cost social activities at senior centers and community centers as effective interventions to decrease social isolation among seniors. There is some evidence that group interventions have been found to be more successful than one-on-one interventions, however more studies are needed before a definite conclusion can be made [11].

We also found that having increased PA was nearly significantly associated with increased social activities. In future studies, we can examine physical activities like swimming, dancing and Tai Chi, which may have not been measured well by the accelerometer-based step counts. In our cohort, woman had more social activities than men. This finding is consistent with several previous studies. One longitudinal study found that social activity remained stable for women as they aged but declined for men [31]; and women had higher levels of leisure activities than men [31]. Such gender differences in the types and levels of social and physical activities will be further examined in our subsequent analyses.

Living alone and age, when adjusted for all other factors in the model, were not associated with social activity frequency. We tend to think of people getting less and less socially active with age, but that may depend on their income, depression, physical disability and ability to drive. To the extent that they can be helped to overcome those challenges, they may remain very active at any age. Fortunately, these potential barriers to social activity appear to be modifiable, suggesting opportunities for future interventions.

We found that direct mail recruitment method was significantly associated with social activities compared to group recruitment, and therefore we included it as a covariable in our analyses. Specifically, participants recruited by direct mail had decreased frequency in social activities compared to participants who were recruited via a group setting. This may be because participants recruited in the group setting may have increased attendance at social activities at baseline. Participant self-selection bias may influence the analytic results. This reinforces the importance of taking recruitment method into account when extrapolating the findings of any study of social behaviors to the general population. This also may suggest that group interventions may be an important avenue to explore when studying areas of intervention to decrease both objective and subjective isolation.

Our study is limited by participant location, cross-sectional nature of the study, and self-reported data. Because of the cross-sectional nature of our study, we cannot predict causation from our data and can only study associations between social isolation, living alone and other factors. As more data are accumulated in our cohort study, we will conduct more thorough longitudinal analysis. Our analysis is also limited by its reliance on participant self-report data. Recall bias, social desirability as well as age-related memory issues may influence the accuracy and reliability of the data analysis. In addition, we sampled neighborhoods through the direct mail campaign in Worcester County, Massachusetts, USA where the demographics of our population were mostly White and suburban. We also recruited participants in limited locations in senior centers, veterans' organizations, and retirement villages. Our cohort is thus not a complete random sample of target population of participants 65 years and older. Despite these weaknesses, our study examined a wide variety of characteristics in older adults, which shed light on the relationships among living status, social support, participation in social activity and healthy aging. The results also suggest that more in-depth analyses of the characteristics of older adults who are socioeconomically disadvantaged, and the specific social support older adults need inside the home are necessary to better understand the determinants of social isolation in order to effectively promote socially-engaged healthy aging.

## 5. Conclusions and future directions

Various factors are associated with social isolation in community-dwelling older adults which are modifiable and important for healthy aging. Interventions specifically increasing instrumental, emotional and informational support in the home and increasing frequency of social activities may lead to better health outcomes and a decreased social isolation in older adults. For example, someone readily accessible for advice from the home, someone to talk to about their worries, and someone to help with household chores may help older adults feel supported and less socially isolated even if they are not able to go into the community as often.

Our data also revealed potential socioeconomic disparities in social isolation, which suggests that continuation of free or low-cost social activities in senior center and community centers may be especially beneficial in poorer communities. We identify several characteristics that may assist in screening for social isolation in older adults including education level, living status, PA, frequency of social activity, and annual income. Older adults with disability, limited income, and lack of transportation may have greater barriers that are associated with increased tendency for social isolation. We also emphasize the importance of considering recruitment method to select target populations for future interventions.

In future studies, we will enrich our cohort to include more rural areas and increase racial and ethnic diversity. In the future, we can also explore how language and cultural differences act as barriers to achieving less social isolation and greater social support. We will also expand our cohort by recruiting at cultural organizations, adult day health care centers, and faith-based organizations. This will ensure that the study results are generalizable to the all populations 65 years old and older. We can further explore how advancements in technology and social media may influence how an older adult perceives their social support and if technology can help with someone who is more socially isolated.
